# TGF Beta Induces Vitamin D Receptor and Modulates Mitochondrial Activity of Human Pancreatic Cancer Cells

**DOI:** 10.3390/cancers13122932

**Published:** 2021-06-11

**Authors:** Camilla Fiz, Giulia Apprato, Chiara Ricca, Alessia Aillon, Loredana Bergandi, Francesca Silvagno

**Affiliations:** Department of Oncology, University of Torino, Via Santena 5 bis, 10126 Torino, Italy; fizcamilla@gmail.com (C.F.); giulia.apprato@edu.unito.it (G.A.); chiara.ricca@unito.it (C.R.); alessia.aillon@gmail.com (A.A.); loredana.bergandi@unito.it (L.B.)

**Keywords:** TGF beta, vitamin D receptor, human pancreatic cancer, epithelial–mesenchymal transition, mitochondria, reactive oxygen species, respiratory chain, cell proliferation, cell motility

## Abstract

**Simple Summary:**

TGFβ is a proinflammatory molecule produced by the tumor and active in its microenvironment. Its influence on cancer metabolism might explain its wide range of effects, but it is still poorly investigated. This study for the first time analyzes the TGFβ/vitamin D interplay in human pancreatic cancer cells and the effects of these two antagonistic molecules on the epithelial–mesenchymal transition, which is an early step of cancer progression. Data collected in this work also reveal the long-term metabolic effects of TGFβ, which are also relevant in the development of pancreatic cancer in vivo. The results of this research suggest that good levels of vitamin D can prevent the early stages of tumor transformation; on the contrary, chronic exposure to the inflammatory cytokine has irreversible consequences since it supports a prometastatic metabolism, confirming TGFβ as a crucial therapeutic target in pancreatic cancer.

**Abstract:**

The inflammatory cytokine TGFβ is both a tumor suppressor during cancer initiation and a promoter of metastasis along cancer progression. Inflammation and cancer are strictly linked, and cancer onset often correlates with the insufficiency of vitamin D, known for its anti-inflammatory properties. In this study, we investigated the interplay between TGFβ and vitamin D in two models of human pancreatic cancer, and we analyzed the metabolic effects of a prolonged TGFβ treatment mimicking the inflammatory environment of pancreatic cancer in vivo. We confirmed the induction of the vitamin D receptor previously described in epithelial cells, but the inhibitory effects of vitamin D on epithelial–mesenchymal transition (EMT) were lost when the hormone was given after a long treatment with TGFβ. Moreover, we detected an ROS-mediated toxicity of the acute treatment with TGFβ, whereas a chronic exposure to low doses had a protumorigenic effect. In fact, it boosted the mitochondrial respiration and cancer cell migration without ROS production and cytotoxicity. Our observations shed some light on the multifaceted role of TGFβ in tumor progression, revealing that a sustained exposure to TGFβ at low doses results in an irreversibly increased EMT associated with a metabolic modulation which favors the formation of metastasis.

## 1. Introduction

Transforming growth factor β (TGFβ) is a pleiotropic cytokine with crucial activity in the regulation of inflammatory responses, which must be accurately restricted in a spatiotemporal way in order to avoid autoimmunity diseases or immune suppression [1]. Indeed, any deregulation of the immune response, such as an uncontrolled condition of chronic inflammation, can be lethal and promote tumorigenesis [2]. In this context TGFβ is both a mediator of the inflammatory response and a well-known inducer of tumor initiation, development, and metastasis formation [3]. TGFβ overexpression has been detected in most types of cancer, and it is a significant marker of aggressive tumor phenotype, associated with poor prognosis [4]. Interestingly, TGFβ has a dual activity since it is an antiproliferative factor, as well as a tumor promoter. With regard to the former, TGFβ is physiologically an inhibitor of cell growth and a proapoptotic factor in nonmalignant cells and damaged tissues, whereby it is highly produced to maintain cellular proliferation homeostasis [4]. On the other hand, in human cancer, TGFβ acts through several complex mechanisms, which drastically depend on the tumor type and the stage of the tumoral development [5]. TGFβ can act as a tumor suppressor and several tumors become resistant to TGFβ, as revealed by many studies that have shown the inactivation of both its receptors (Tβ-RI and Tβ-RII) in numerous human cancer cell lines, including colon and gastric cancer [6]. Furthermore, TGFβ protumoral activity is significant both inside cancer cells and in their proximity, which includes immune cells, endothelial cells, and stromal fibroblasts as target cells. The paracrine activity favors the development of a microenvironment that improves the tumor survival, invasiveness, and growth [7]. Indeed, TGFβ promotes the basal detachment of cancer and endothelial cells by inducing metalloproteinases expression, secretion, and activity. Furthermore it enhances the epithelial-to-mesenchymal transition (EMT), downregulating E-cadherin (E-cad), a crucial invasion suppressor and cell-adhesion receptor [7]. Therefore, TGFβ is supposed to have a prometastatic function by enhancing cancer cell spread, with a further direct prometastatic activity on specific tissues (e.g., lung parenchyma in breast cancer) [8]. Moreover, TGFβ and its signaling pathway have a strong profibrogenic activity that stimulates the deposition of extracellular matrix proteins (e.g., collagen and elastin) in the interstitial fluid, impairing the tissue function and differentiation [9].

The influence of TGFβ on cellular metabolism can drive the cell toward the metabolic shift required for cancer growth. In fact, substantial evidence has recently demonstrated that TGFβ drastically affects the metabolism of cancer and neighboring cells. In particular, changes in the lipid metabolism and in metabolic reprogramming, as well as their consequences for mitochondrial activity, are attributed to TGFβ [10,11]. With regard to the latter, a few studies have supported the strong effects of TGFβ on mitochondria through several mechanisms, although metabolic control can lead to opposite results. In human lung fibroblasts, the SMAD-dependent pathway activated by TGFβ upregulates the mitochondrial mass with the consequent progression of fibrosis [12], as well as controls the mitochondrial biogenesis in adipocytes of obese and diabetic mice [13]. Moreover, TGFβ can directly modulate the mitochondrial metabolism by affecting the activity of the electron chain transport (ECT) components and the mitochondrial function. Some lines of evidence show that TGFβ impairs the ATP-coupled respiration with the inhibition of complexes V [14] and IV and a reduction in mitochondrial potential [15]. However, TGFβ can also boost the mitochondrial activity of fibroblasts, the ATP/ADP ratio, and mitochondrial respiration, inducing their activation into myofibroblasts [16]. Furthermore, in our previous studies, we reported a significant increase in mitochondrial membrane potential and in transcript expression of subunit 2 of cytochrome C oxidase (COX-2) on human bronchial epithelial cells incubated with TGFβ [10]. In addition, the significant increase in ATP levels and ROS production in treated cells further demonstrates the capacity of TGFβ to induce mitochondrial respiration [10]. Altogether, these data reveal the multifaceted activity of TGFβ and suggest that its metabolic effects are strongly context-dependent.

Free radicals such as ROS (reactive oxygen species) are key molecular players in linking inflammation to cancer [17,18,19]. As for TGFβ-mediated ROS production, it is univocally accepted by the scientific community that TGFβ is responsible for redox imbalance and oxidative stress [20,21]. Indeed, TGFβ promotes the production of reactive oxygen or nitrogen species (ROS/RNS) higher than in physiologic conditions, which can lead to redox imbalance and apoptosis [22]; in addition, ROS are essential modulators of fibrosis, gene expression, and cellular motility, and they deeply influence the tumor environment [23].

Considering this background, the research of therapies targeting the protumoral and pro-oxidant effects of TGFβ could be a crucial starting point to contrast the development of both cancer and microenvironmental inflammation. Notably, a recent study revealed that the metabolic effects of TGFβ are reverted by the vitamin D signaling pathway [10], which has a central role in maintaining the differentiated state of tissues. By binding the vitamin D receptor (VDR), the active form of vitamin D opposes the EMT response. In fact, it induces the expression of numerous antitumoral genes, increasing the levels of E-cad and cystatin D, while contrasting the Wnt/β catenin pathway. Moreover, for the first time, Ricca et al. [10] demonstrated the reciprocal regulation between TGFβ and the vitamin D/VDR activity in human models of EMT. The results of this study demonstrated that TGFβ is able to induce the expression of VDR, while VDR exerts a negative feedback on TGFβ signaling by inhibiting TGFβ-mediated metabolic effects. Indeed, activation of the VDR signaling pathway triggered by TGFβ suppresses mitochondrial energy metabolism, ROS production, and cell migration.

Considering the heterogeneous effects of the cytokine, a more comprehensive analysis of TGFβ activity in tumoral cells is required, and the TGFβ–VDR feedback must be further demonstrated in cancer. For this purpose, in the present study, we investigated two human pancreatic cancer cell lines and we found them sensitive to the TGFβ–VDR interplay, corroborating the relevance of these signaling pathways in tumor prevention. Moreover, we explored the metabolic effects of a prolonged exposure to TGFβ with the intent of elucidating its contrasting activities in cancer cells, which are surrounded in vivo by an inflammatory microenvironment. The results of this work reveal that a prolonged exposure to TGFβ at low doses induces EMT together with a metabolic modulation which favors the motility of cancer cells.

## 2. Results

### 2.1. The Response of Two Human Pancreatic Cancer Models to TGFβ and Vitamin D and the Modulation of Vitamin D Receptor

**Figure 1:**
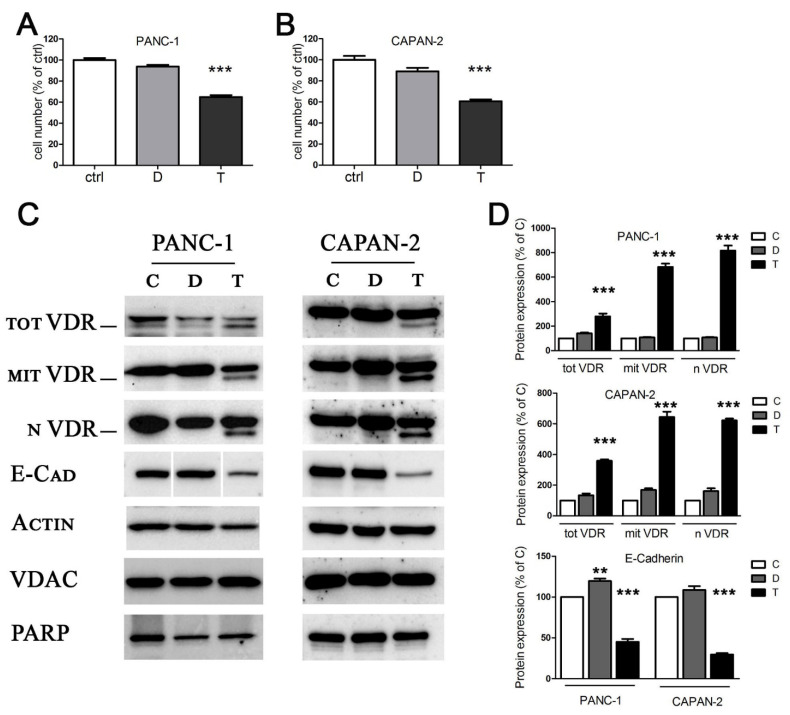
The effects of TGFβ and vitamin D on proliferation and protein expression. (**A**,**B**) After 5 days of treatment with 100 nM 1,25(OH)_2_D_3_ (D) and 10 ng/mL TGFβ (T), PANC-1 and CAPAN-2 cell proliferation was measured by crystal violet staining, and the values are expressed as percentages of the untreated control. (**C**) After 48 h of treatment, the Western blot analysis revealed the expression of total, mitochondrial, and nuclear VDR, and the expression of the epithelial marker E-cadherin. Actin, VDAC, and PARP were used as loading controls for total, mitochondrial, and nuclear extracts, respectively. Original Western blots are shown in Figures S1 and S2 (Supplementary Materials). (**D**) Bands were quantified and normalized by loading control bands on the same blot. Data expression is relative to control. Graphs represent the means ± SEM. ** *p* < 0.01 vs. ctrl; *** *p* < 0.001 vs. ctrl.

We selected two human pancreatic cancer cell lines widely exploited by in vitro tumoral studies [24,25]: PANC-1 and CAPAN-2 cells. They were both derived from primary tumors and they are representative of a different tumoral aggressiveness. In this regard, CAPAN-2 cells are characterized by a less aggressive phenotype, since they are well differentiated and do not induce metastasis; on the other hand, the poorly differentiated and strongly metastatic PANC-1 cell line is representative of an aggressive phenotype of human pancreatic cancer [26]. Many cancers (indeed, pancreatic cancer is no exception) are resistant to the antiproliferative and differentiating properties of vitamin D, and pancreatic cancer cells often show low levels of its receptor [27]. The sensitivity of the two cell lines to TGFβ and to the active form of vitamin D (1,25(OH)_2_D_3_, henceforth named vitamin D) was tested after five days of treatment. CAPAN-2 and PANC-1 were incubated with 100 nM 1,25(OH)_2_D_3_ or 10 ng/mL TGFβ, which are the standard conditions usually employed for in vitro studies of EMT and signal transduction [28,29,30]. We confirmed the tumor-suppressive activity of TGFβ on this cancer model, because treatment with the cytokine reduced the number of cells as detected by crystal violet staining, due to cytotoxicity or inhibition of growth. On the other hand, the antiproliferative effect of vitamin D was modest and not significant, as shown in Figure 1A,B, thus suggesting a resistance to the hormone. Next, we investigated the expression of vitamin D receptor (VDR), since its epigenetic silencing could explain the lack of effect of the treatment with vitamin D. In our previous studies, we demonstrated the expression of VDR in total, mitochondrial, and nuclear fractions of several cell models [31,32,33]. In pancreatic cancer cells, in accordance with the poor sensitivity to vitamin D, neither cell line expressed the receptor when untreated, and the absence was evident in both the nuclear and the mitochondrial compartments in which the receptor is active [31,32] (Figure 1C). Most interestingly, we discovered the induction of VDR in both compartments after stimulation with TGFβ (Figure 1C, quantified in Figure 1D); this observation, novel in the pancreatic model, confirmed the interplay between 1,25(OH)_2_D_3_ and TGFβ, whereby TGFβ induces VDR expression, previously demonstrated by our studies on human lung epithelial cells [10]. The analysis of protein expression levels revealed that the treatment with TGFβ effectively drove the cells toward EMT, as suggested by the reduction in E-cadherin (Figure 1C, quantified in Figure 1D). Original blots are shown in Figures S1 and S2 (Supplementary Materials).

Altogether, these data led to the identification of two models of human pancreatic cancer responsive to TGFβ and possibly sensitive to vitamin D, but only as a consequence of the treatment with the cytokine, due to VDR induction; these models can be used to further corroborate the 1,25(OH)_2_D_3_/TGFβ interplay in tumoral cells, and we explored this aspect in further experiments.

### 2.2. Vitamin D Opposes the Epithelial–Mesenchymal Transition Induced by TGFβ

**Figure 2:**
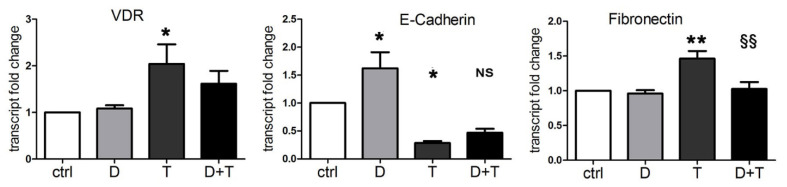
Vitamin D reduces the EMT induced by TGFβ. RT-PCR assay evaluated the expression of VDR, E-cadherin, and fibronectin transcripts. PANC-1 cells were incubated for 48 h with single 100 nM 1,25(OH)_2_D_3_ (D) and 10 ng/mL TGFβ (T) or combined (D + T) treatments. The plotted values represent the fold change relative to untreated cells. Data are displayed as the means ± SEM of three independent experiments. ns: not significant vs. ctrl; * *p* < 0.05 vs. ctrl; ** *p* < 0.01 vs. ctrl; ^§§^ *p* < 0.01 vs. T.

It is well known that vitamin D contrasts EMT in normal and cancer cells, by inducing cell differentiation and increasing cell adhesion structures [34]. Since we discovered that TGFβ was able to induce the expression of VDR, we wondered whether vitamin D could be effective in reducing TGFβ-triggered transition, even in our cancer models insensitive to the antiproliferative action of the hormone. Therefore, we verified the effects on EMT of 1,25(OH)_2_D_3_ and TGFβ, alone or combined, by measuring the transcription of EMT markers: epithelial E-cadherin and mesenchymal fibronectin. In this set of experiments, the induction of VDR by TGFβ was confirmed at the transcriptional level, as shown in Figure 2. Moreover, we found that vitamin D increased E-cadherin transcription, and that TGFβ decreased E-cadherin and increased fibronectin transcription, as expected. Interestingly, when vitamin D was added to TGFβ treatment, it reduced the effects of the cytokine; as shown in Figure 2, the cotreatment brought the transcription of E-cadherin back to levels similar to the control (difference not significant between D + T and ctrl). Moreover, the cotreatment reverted the increase in the mesenchymal marker (significant difference between D + T and T for fibronectin).

These data are in line with previous considerations based on our studies and others [10,30], demonstrating that TGFβ promotes the EMT phenotype and vitamin D counteracts the transition. For the first time, we demonstrated that, due to the induction of VDR, vitamin D was able to oppose the EMT induced by TGFβ in a pancreatic cancer model.

### 2.3. Characterization of TGFβ Metabolic Effects in Pancreatic Cancer Cells: Dose and Time Dependency

**Figure 3:**
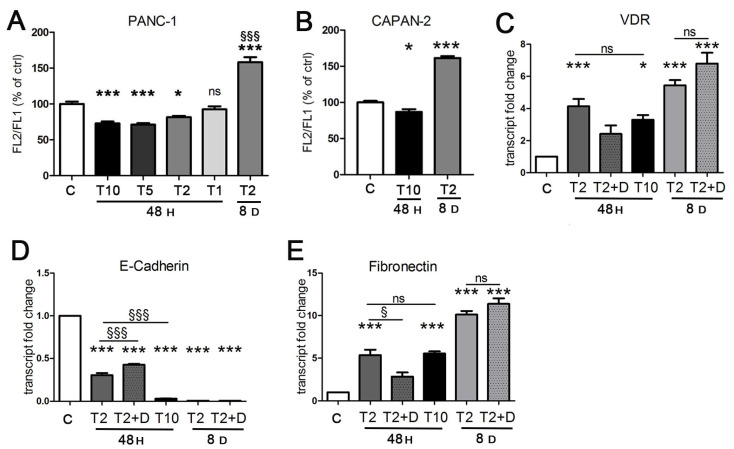
The effects of TGFβ treatment are dose- and time-dependent. Cells were incubated for 48 h with TGFβ at 10 ng/mL (T 10), 5 ng/mL (T5), 2 ng/mL (T2), and 1 ng/mL (T1), and for 8 days with TGFβ at 2 ng/mL, and compared to untreated samples (C). (**A**,**B**) The mitochondrial membrane potential of PANC-1 and CAPAN-2 cells was assessed by cytofluorimetric evaluation of JC-1 dye. The FL-2/FL-1 ratio was calculated and is expressed as a percentage of the value obtained for untreated cells. (**C**–**E**) The transcriptional activity of TGFβ alone or in combination with 100 nM 1,25(OH)_2_D_3_ in the last 2 days of incubation (T2 + D) was evaluated in PANC-1 cells by RT-PCR analysis. The plotted values represent the fold change in transcript expression relative to untreated cells. Data are displayed as the means ± SEM of three independent experiments. ns: not significant vs. ctrl or vs. the indicated sample; * *p* < 0.05 vs. ctrl; *** *p* < 0.001 vs. ctrl; ^§^ *p* < 0.05 vs. the indicated sample.; ^§§§^ *p* < 0.001 vs. T2 48 h or vs. the indicated sample.

In the first experiments, we confirmed the short-term transcriptional activity of TGFβ in EMT, unveiling its important link with the vitamin D axis. Next, we sought to investigate the metabolic effects of the cytokine which could contribute to cancer progression. Considering that a chronic inflammatory microenvironment promotes pancreatic cancer in vivo [35], we decided to explore both short and long exposure to TGFβ. We tested different doses of the cytokine and we considered both short and long treatments. We analyzed the mitochondrial metabolic changes in response to TGFβ treatment starting from an evaluation of the mitochondrial activity rate. For this purpose, we performed a cytofluorimetric analysis with JC-1 dye to measure the mitochondrial membrane potential, which is proportional to mitochondrial respiratory activity. As shown in Figure 3A, we observed a drastic decrease in mitochondrial membrane potential when PANC-1 cells were treated with 10 ng/mL TGFβ (T10) for 48 h, compared to the untreated control, which suggested the cellular condition of stress caused by the acute treatment. As reported in Figure 3A, the sensitivity of mitochondrial activity to TGFβ treatment was dose-dependent, because the decrease in TGFβ concentration corresponded to an increase in mitochondrial potential, until it was quite similar to the untreated control. Considering these data, we selected the concentration of 2 ng/mL (T2) as the safest but still effective dose of TGFβ and we used this concentration to evaluate the effects of a prolonged treatment while minimizing toxicity. The exposure of PANC-1 at this dose for 8 days produced a strong increase in mitochondrial respiratory activity. In agreement with these results, after the short-term exposure (10 ng/mL TGFβ for 48 h), CAPAN-2 cells also decreased their mitochondrial membrane potential, which was instead drastically boosted in response to the long-term exposure (2 ng/mL for 8 days) compared to the control (Figure 3B). To verify the effects of TGFβ on cell viability and its potency, we calculated the EC_50_ after both 48 h and 8 days of treatment. In PANC-1 cells, we estimated a value of 35 ng/mL when the EC_50_ for TGFβ was calculated after 48 h of treatment and an EC_50_ of 8 ng/mL when the cytokine was used for 8 days.

These results persuaded us that the approach of a diverse treatment could provide important information about the metabolic impact of TGFβ depending on the context. Therefore, we decided to proceed with the investigation by evaluating both the short exposure to a high concentration (T10) commonly used in EMT studies in vitro [28,29,30] and the prolonged exposure to the lower concentration (T2) that allows longer treatments without damaging the cancer cells.

Because the mitochondrial metabolic effects triggered by TGFβ were opposite depending on context, we verified that TGFβ maintained its transcriptional activity even at low doses after prolonged exposure. We found that this was the case; indeed, the induction of VDR and the transcriptional control of EMT markers was identical, even potentiated, at day 8 compared to 48 h (Figure 3C–E). In fact, the effect of T2 at 8 days was stronger than T2 and T10 at 48 h. We did not test the higher T10 dose after 8 days because the EC_50_ assay revealed a cytotoxicity of this concentration incompatible with a correct evaluation of metabolic parameters or gene expression. Although after 48 h vitamin D was able to refrain the efficacy of TGFβ (Figure 2), the hormone failed to revert the transcriptional effects of TGFβ when it was added at the end of long-term incubation (Figure 3C–E). We concluded that vitamin D lost its power to revert the transition when it was added in the last phase of TGFβ exposure, and this was not surprising because it confirmed similar observations obtained in a previous investigation on epithelial cells [10].

### 2.4. The Cytotoxic Production of ROS Triggered by TGFβ Is Abated at Low Concentration of the Cytokine

**Figure 4:**
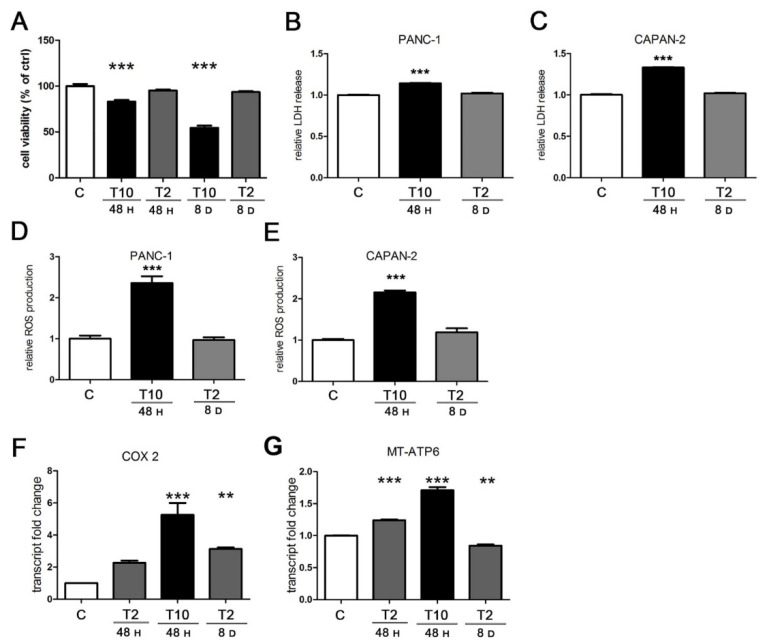
TGFβ induces cytotoxicity, ROS production, and mitochondrial transcription. PANC-1 and CAPAN-2 cells were exposed to short- and long-term treatments as previously described. (**A**) Cell viability was measured by MTT assay and expressed relative to control. (**B**,**C**) Cytotoxicity was measured by the LDH release in the culture medium and expressed relative to control. (**D**,**E**) Intracellular ROS levels were assayed by the measurement of DCF fluorescent emission and results expressed relative to control. (**F**,**G**) PANC-1 cells were analyzed by RT-PCR for COX-2 and MT-ATP6 transcript expression. The values plotted on the graph represent the fold change in transcript expression in treated versus untreated cells. Data are displayed as the means ± SEM of three independent experiments.; ** *p* < 0.01 vs. ctrl; *** *p* < 0.001 vs. ctrl.

A reduction in mitochondrial potential is one of the most evident signs of cellular stress; therefore, we hypothesized that high doses of TGFβ could cause cell damage. For this reason, we evaluated the cytotoxicity caused by TGFβ at different conditions using the MTT viability assay and lactate dehydrogenase (LDH) release assay. As expected, the viability test demonstrated the cytotoxicity of T10 at 48 h and even more after 8 days, whereas T2 never affected cell integrity (Figure 4A). The toxicity of T10 was confirmed by the LDH release assay; in fact, PANC-1 cells responded to the acute treatment (T10 for 48 h) with a relative LDH release higher than the untreated control. On the contrary, the prolonged treatment (T2 for 8 days) was well tolerated and was characterized by a release similar to the control (Figure 4B). The difference was confirmed in CAPAN-2 cells, which were even more sensitive to the acute toxic TGFβ exposure (Figure 4C).

Given the toxicity of TGFβ treatment at high doses, we sought to investigate the cellular process mainly involved. In this regard, it has been widely demonstrated that toxicity is related to an excessive ROS production [9]; indeed, the production of ROS triggered by TGFβ is well known and it occurs through many mechanisms, such as the impairment of the mitochondrial function and the suppression of antioxidant enzymes [36]. Considering the above, we measured the production of ROS in PANC-1 and CAPAN-2 cells under acute (10 ng/mL for 24 h) and prolonged (2 ng/mL for 8 days) TGFβ treatment. As shown in Figure 4D, we found a strong augment in ROS levels in response to the acute exposure, compared to control. Interestingly, ROS production was not modulated by the long-term exposure to TGFβ. The same results were obtained in CAPAN-2 cells (Figure 4E).

An increased production of ROS could have a mitochondrial origin; for example, it could be elicited by a deregulated respiratory activity. Indeed, it has been reported that TGFβ can modulate the transcription of the electron transport chain (ETC) [37]; therefore, we evaluated the transcriptional effects of TGFβ on mitochondrial respiration. By RT-PCR, we assessed the expression levels of two mitochondrially encoded mRNAs of PANC-1 cells: cytochrome C oxidase subunit 2 (COX-2) and ATP synthase membrane subunit 6 (MT-ATP6). Compared to the control, the short-term exposure triggered a fivefold induction of COX-2 at T10 and a twofold increase at T2, although the latter was not significant after statistical analysis. We also found a mild induction (threefold) after chronic exposure at T2 (Figure 4F). T10 was not tested after 8 days because of its toxicity. As for the MT-ATP6 transcript, we detected a relevant increase in response to the acute TGFβ treatments, but not after a prolonged incubation (Figure 4G).

These data supported the conclusion that the toxicity of the acute treatment with TGFβ, detected both by viability assay and by LDH release, associated with the decrease in mitochondrial potential, was due to the increased ROS production, whereas the prolonged TGFβ treatment at low doses did not damage the human pancreatic cancer cells because ROS levels did not change. Moreover, we observed that ROS production occurred together with a striking transcriptional induction of respiration, whereas ROS levels did not change when the transcriptional modulation was modest. Altogether, these data reveal the drastic effects exerted by TGFβ on cellular metabolism and health, because the cytokine affects mitochondrial activity with a dose-dependent efficacy, leading to a proportional production of ROS and a consequent impact on cell survival.

### 2.5. The Long-Term TGFβ Treatment at Low Doses Promotes Cell Migration

**Figure 5:**
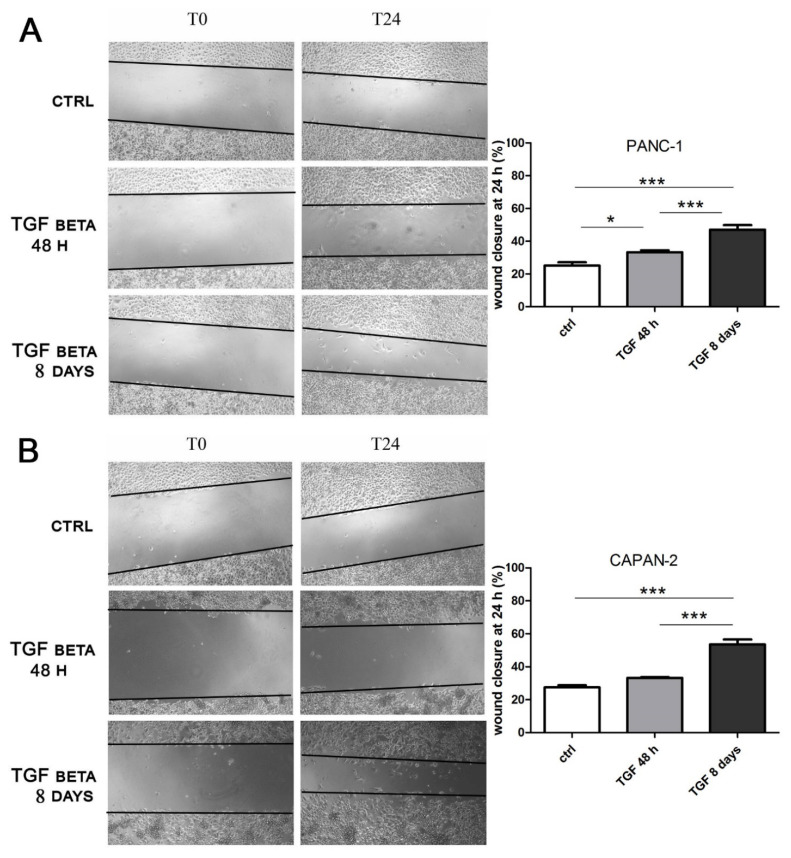
The long-term treatment with TGFβ at low doses promotes cell migration. PANC-1 (**A**) and CAPAN-2 (**B**) cells were incubated with TGFβ at 10 ng/mL for 24 h or at 2 ng/mL for 7 days, and then the treatment was continued for another 24 h to carry out the wound healing assay. The effects on cell migration at time 0 (T0) and after 24 h from scratch (T24) were evaluated using a wound-closure assay. The figure presents the empty areas in the wound-closure assay under different experimental conditions; these areas were measured and expressed on the graph as a percentage of wound closure. Data are displayed as the means ± SEM. * *p* < 0.05; *** *p* < 0.001.

As the last step of our analysis, we decided to investigate the impact of short- and long-term exposure to a different concentration of TGFβ on another crucial marker of cancer progression, which is cancer cell migration. PANC-1 and CAPAN-2 cells were analyzed by the wound healing cell migration assay after treatment with TGFβ at high doses (10 ng/mL) for 24 h and at low doses (2 ng/mL) for 8 days. As shown in Figure 5, for both cancer cell lines, the wound closure after short-term treatment was modest and similar to the control, despite the induction of EMT demonstrated by the first experiments of this study; however, after prolonged exposure, the treated cells showed a remarkable ability to migrate (Figure 5), significantly higher than the movement observed in untreated cells or after the short-term treatment. These data confirm the drastic effect of a prolonged TGFβ treatment in promoting cancer cell motility and, for the first time, show that the response is dose- and time-dependent.

## 3. Discussion

TGFβ is a central player in inflammation. Its protumoral effects can explain why the inflammatory status can predispose to the onset of cancer. It must be emphasized that the activity of the cytokine has multiple, even contrasting consequences, depending on the context. While signaling pathways and transcriptional activity have been described in detail and are largely studied, little is known about the metabolic effects of the cytokine. The modulation of cellular metabolism can lead to a context-dependent outcome; therefore, the investigation of the metabolic effects exerted by TGFβ can shed some light on the conflicting results of its activity.

In this study, we investigated the effects of a chronic inflammatory stimulus on two models of pancreatic cancer, and we described the impact of vitamin D and the relevance of TGFβ metabolic activity on cancer progression. Vitamin D shows differentiating and anti-inflammatory properties [38,39], and its efficacy on cancer could depend on its influence on TGFβ signaling. We chose pancreatic cancer as the target of our analysis for several reasons. First of all, it is a cancer that develops through a mesenchymal transition phase with a deadly metastatic spread [40,41]; thus, it allows considering the influence of TGFβ on both aspects of the progression. Moreover, it is a highly fibrotic cancer, as the result of the abundant production of TGFβ by cancer cells and due to its autocrine and paracrine activity [42,43]. The extensive fibro-inflammatory stroma of pancreatic ductal adenocarcinoma plays an active role in its progression and therapeutic resistance, highlighting the importance of acquiring more details on TGFβ activity in this type of cancer. Furthermore, vitamin D deficiency is often associated with cancer development, and it may play a role in both incidence and survival from pancreatic cancer [44,45]; therefore, revealing the efficacy of the hormone in contrasting TGFβ activity could explain the link. Indeed, the first findings of this study confirmed the interplay between TGFβ and vitamin D previously reported only on epithelial cells [10]. For the first time, we demonstrated in human pancreatic cancer the induction of VDR and we revealed that the upregulation of VDR mediated the feedback mechanism via which vitamin D can counteract the EMT induced by TGF beta. Interestingly, we found that vitamin D lost its efficacy when added after a prolonged incubation with TGFβ, in agreement with previous conclusions that the beneficial power of vitamin D is related to the early stages of TGFβ-driven transition [10]. The findings of the present study are relevant considering that proper levels of vitamin D could be important for pancreatic cancer prevention; in fact, it is conceivable that vitamin D could act as a tamer of the excessive activity of TGFβ and could avert cancer.

In this study we found that the metabolic effects of TGFβ were related to dosage and exposure time. In our in vitro models of pancreatic cancer, the cytokine was cytotoxic at the highest doses of 10 ng/mL and especially after prolonged exposure, due to the strong stimulation of respiratory chain associated with the overproduction of ROS, which in turn damaged mitochondrial membrane potential. It is of note that these concentrations are normally used in in vitro studies exploring EMT [34,35,36]. Our observation that the high concentration of TGFβ affects viability on long-term treatments is not in contrast with data from other studies, because the most common experimental setup in EMT investigation is the treatment with 10 ng/mL TGFβ only for short EMT induction (up to 48 h in pancreatic cancer [46,47,48]), with a lower concentration for longer induction (for example, breast cancer [49,50,51,52] and melanoma cancer [53]). To our knowledge, the effects of prolonged treatments at high doses have never been reported. In our model, we estimated an EC_50_ of 35 ng/mL and 8 ng/mL when TGFβ was used for 48 h and 8 days, respectively. On the other hand, the cancer cells adapted to lower doses, and, after a prolonged exposure, the metabolic effect of TGFβ as an inducer of respiratory activity became evident. In fact, the low levels of TGFβ stimulated a moderate induction of the respiratory chain, which did not trigger the production of ROS and led to an increase in mitochondrial membrane potential, thus leaving the cell healthy and supported in its motility. The mitochondrial synthesis of ATP is of paramount importance for migration taking place in EMT, as several studies have demonstrated [54,55,56]; therefore, we concluded that the moderate, nontoxic enhancement of mitochondrial activity supported cancer spread. Again, our analysis of cancer cell motility revealed that the response to TGFβ was dose-dependent. In fact, in agreement with previous studies [46], the most used treatment with high dose and short time had little effect on pancreatic cancer motility, despite inducing EMT markers; however, upon prolonged exposure to a lower concentration, the motility was strongly increased; this different dose-dependent response has never been underlined before.

**Figure 6:**
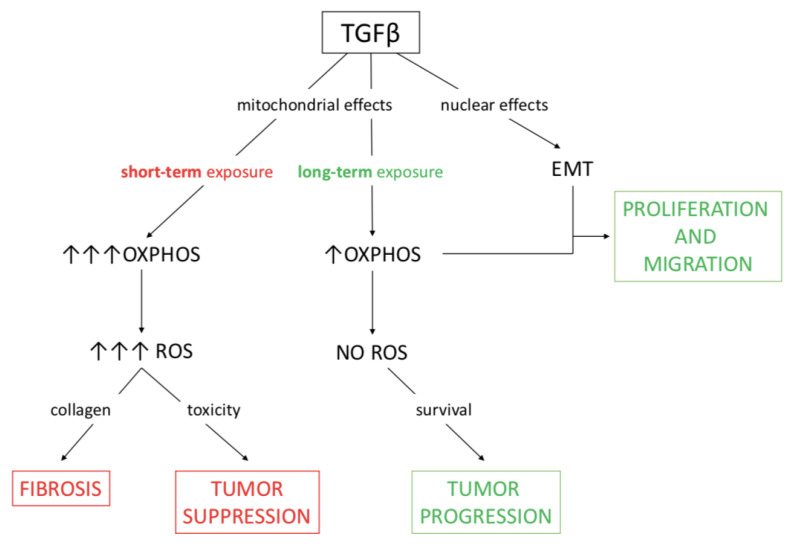
A working model of the metabolic effects of TGFβ which sustain either tumor suppression or tumor progression. The consequences of TGFβ activity on mitochondrial respiration can be either tumor suppression or fibrosis when ROS production is high, whereas tumor progression takes place when ROS levels are checked. In the investigated model of pancreatic cancer extensively treated at low doses of TGFβ, metabolic and nuclear effects of the cytokine together support EMT and then migration.

The transcriptional control exerted by TGFβ was evident at both high and low concentration of the cytokine, which activated the nuclear program driving the EMT in all conditions. However, the different impact on metabolism could, at least in part, explain the heterogeneous response of cancer cells to the cytokine. Actually, other studies have described TGFβ as both an inhibitor of mitochondrial metabolic activity [15,57] and an inducer of respiratory oxidative metabolism [58,59]; similarly, the dual role of TGFβ in tumors is also well known, since it acts as both a suppressor and a promoter of cancer progression. The evidence found in our study supports both roles for the cytokine, depending on a context-dependent influence on mitochondrial metabolism. Moreover, the metabolic effect exerted on cancer-associated fibroblasts and the consequent increased production of ROS would lead to collagen secretion and fibrosis; this consideration warrants further investigation. The data obtained in this work highlight the dual impact of TGFβ on mitochondrial metabolism and production of ROS, which can oppose or sustain EMT induced at the transcriptional level; such conclusions are depicted in Figure 6.

The approach of investigating in vitro the activity of TGFβ considering the long-term effects of the cytokine has the merit of assessing the results of a chronic stimulation, which mimics the inflammatory condition considered in vivo a protumorigenic stimulus. TGFβ is produced by both cancer cells and stromal tissue, in addition to the molecules of systemic origin. Therefore, measuring its levels in the tumoral site in vivo is complex and untested. Moreover, cancer cells are often resistant to TGFβ action, and the final effect of the cytokine in vivo could be comparable to that observed in vitro at low doses. In our in vitro model, we started from the standard dose used in most studies of EMT, and we found that the lower concentration of 2 ng/mL was the best compromise between efficacy and lack of toxicity after prolonged exposure; however, long-term incubation could be carried out at different dosage in other cellular models. The recent and very elegant work of Schworer et al. demonstrated that TGFβ at the same concentration of 2 ng/mL induces mitochondrial respiration and collagen synthesis in fibroblasts, without production of ROS [58]. The response of cancer cells to inflammatory signals could be influenced by their mutation status; depending on its genetic background, each tumor can respond to external stimulation differently. As for TGFβ, we showed that the production of ROS could explain the anticancer properties of high doses of the cytokine; therefore, the efficiency of the intracellular antioxidant defenses could determine the results of TGFβ activity. Mutated K-RAS increases glutamine utilization, and glutamine metabolism is used by cancer cells to produce NADPH essential in antioxidant defense [60]. Moreover, glutamine can also be diverted toward proline biosynthesis and, again, this alternative pathway represents an antioxidant strategy [58]. Pancreatic cancer characterized by mutated K-RAS is, therefore, protected from oxidative stress and takes advantage from the transcriptional activity of TGFβ driving EMT. Other mutations or specific differentiation status of different pancreatic cancer subpopulations could have similar protective effects from the anticancer activity of TGFβ.

## 4. Materials and Methods

### 4.1. Cell Culture and Treatments

The human pancreatic cancer cells PANC-1 and CAPAN-2 were bought from American Type Culture Collection (ATCC), USA. They were grown at 37 °C in humified 5% CO_2_ atmosphere in DMEM culture medium supplemented with 10% fetal bovine serum and 1% antibiotics (penicillin/streptomycin (Sigma-Aldrich, St. Louis, MO, USA)). Cells were stimulated with 100 nM 1,25(OH)_2_D_3_ (Sigma-Aldrich, St. Louis, MO, USA) and TGF-β1 at different doses (PeproTech, Rocky Hill, NJ, USA) for 48 h or 8 days.

### 4.2. Cell Proliferation Assay

PANC-1 and CAPAN-2 cells were seeded in 96-multiwell plates and were incubated with 10 ng/mL TGFβ and 100 nM 1,25(OH)_2_D_3_. After the treatment, they were fixed with 11% glutaraldehyde for 15 min, and then cells were stained with 0.1% crystal violet solution for 20 min, followed by solubilization with 10% acetic acid solution [10]. The absorbance of crystal violet was recorded at 595 nm, and the data were collected as the average of three independent experiments for each experimental condition.

### 4.3. Extract Preparation and Western Blotting Analysis

The subcellular fractionation was carried out as previously reported [10,31,32]. Cells were scraped from the culture dishes and homogenized in lysis buffer containing 10 nM Tris-HCl at pH 7.4, 0.33 M saccharose, 1 mM EDTA, 1 mM dithiothreitol, 1 mM phenylmethylsulphonyl fluoride (PMSF), 1 mM proteases inhibitor Cocktail set III (Calbiochem), 1 nM Na_3_VO_3_, and 1 mM NaF. Lysates were subjected to differential centrifugation to isolate the nuclear and mitochondrial fraction. Western blot analysis of 30 µg of total lysate, as well as of mitochondrial and nuclear fractions, was carried out after separation by SDS-PAGE at 10%. Proteins of interest were detected with the following antibodies: mouse monoclonal antibodies anti-VDR (sc-13133) and anti E-cadherin (sc-21791) purchased from Santa Cruz, CA, USA. The loading controls were checked on the same membranes and detected by antibodies anti-VDAC (monoclonal anti-porin 31HL, Calbiochem), anti-actin (mouse monoclonal sc-8432 Santa Cruz), and rabbit antibody anti-PARP (sc-7150, Santa Cruz). The bands relative to actin, VDAC, and PARP were chosen as proteins enriched in total, mitochondrial, and nuclear fractions, respectively, and were used as references to correct the quantification of the proteins of interest. The correct band corresponding to VDR was identified in past studies by molecular weight and silencing experiments [31,32], and it corresponds to the lower band when a doublet band is present, whereas the upper band is due to unspecific labeling. The signal that detects VDR is indicated in all figures.

### 4.4. Real-Time Polymerase Chain Reaction (qRT-PCR)

TRIzol (Invitrogen, Thermo Fischer Scientific, Waltham, MA, USA) was used for the extraction of total RNA of which 1 µg was retrotranscribed in cDNA by the High-Capacity cDNA Reverse Transcription kit (Thermo Fisher Scientific, Waltham, MA, USA). The quantitative PCR was carried out using the kit iTaqTM Universal SYBR^®^ Green Supermix (Bio-Rad, Hercules, CA, USA) and the following primers: VDR fwd 5′–ACTTGTGGGGTGTGTGGAGAC–3′, rev 5′–GGCGTCGGTTGTCCTTCG–3′; E-cadherin fwd 5′–TACGCCTGGGACTCCACCTA–3′, rev 5′–CCAGAAACGGAGGCCTGAT–3′; fibronectin fwd 5′–GTGCCTGGGCAACGGA–3′, rev 5′–CCCGACCCTGACCGAAG–3′; COXII fwd 5′–TCTGGTCAGCCCAACTCTCT–3′, rev 5′–CCTGTGATCCACCAGAAGGT–3′; MT-ATP6 fwd 5′–CCAATAGCCCTGGCCGTAC–3′, rev 5′–CGCTTCCAATTAGGTGCATGA–3′; S14 fwd 5′–AGGTGCAAGGAGCTGGGTAT–3′, rev 5′–TCCAGGGGTCTTGGTCCTATTT–3′.

The housekeeping gene ribosomal subunit protein S14 was used as an internal control. The PCR required one denaturation cycle at 95 °C for 2 min, 40 amplification cycles with denaturation at 95 °C for 15 s, and then 30 s of annealing/extension at 60 °C. Data were analyzed according to the 2^−ΔΔCt^ method.

### 4.5. Measurement of Mitochondrial Membrane Potential (Δψm)

JC-1 (5,5′,6,6′-tetrachloro-1,1′,3,3′-tetraethylbenzimidazolylcarbocyanine iodide) is a mitochondrial and cationic dye that accumulates in the mitochondria of living cells in a membrane potential-dependent manner. JC-1 is soluble in cytoplasm with a green fluorescent emission (530 nm), and it aggregates and shifts to red (590 nm) in mitochondria.

The assay was carried out as previously described [10]. Briefly, treated cells were harvested by trypsinization and loaded with JC-1 (2 µg/mL final concentration). Intracellular JC-1 was quantified at 530 nm (FL-1 green fluorescence) and 590 nm (FL-2 red fluorescence) by flow cytometry. The ratio of FL2/FL1 was evaluated to determine ΔΨm. Experiments were performed in triplicate and repeated three times.

### 4.6. Intracellular ROS Production

With the aim of evaluating a general production of radical species as the byproducts of mitochondrial respiratory stimulation, we employed the most widely used probe 2′,7′-dichlorodihydrofluorescein diacetate (DCFH-DA), which is used to investigate redox signaling mechanisms and the generation of oxidants [61]. DCFH-DA is a membrane-permeable probe which is transformed into DFCH by cytoplasmic nonspecific esterases and is then oxidized by reactive oxygen species (ROS), becoming the fluorescent dichlorofluorescein (DCF). The assay was carried out as previously reported [10]; briefly, after treatments, cells were incubated with 10 μM (DCFH-DA, Sigma) for 15 min. Fluorescence intensities were measured (excitation wavelength of 504 nm and emission wavelength of 529 nm) by a Packard EL340 plate reader (Bio-Tek Instruments, Winooski, VT, USA); fluorescent values were normalized to the protein content and expressed as values relative to control. Experiments were performed in triplicate and repeated three times.

### 4.7. Cell Viability Assay

Cells were treated with TGF beta at different concentrations, from 0.1 ng/mL to 100 ng/mL, or left untreated as control for either 48 h or 8 days. Then, cells were incubated with 500 µg/mL methylthiazolyldiphenyl-tetrazolium bromide (MTT, Merck, Milan, Italy) in serum-free DMEM for 2 h at 37 °C according to Mosmann [62]. Finally, the formazan crystals formed in the bottom of the plate were dissolved with DMSO and the absorbance at 550 nm was measured in a Packard EL340 microplate reader (Bio-Tek Instruments, Winooski, VT, USA). The 50% effective concentration (EC_50_) value was determined using GraphPad Prism software (version 6.01, San Diego, CA, USA).

### 4.8. Toxicity Test (LDH Release)

After treatments, the cellular damage was measured by lactate dehydrogenase (LDH) release in the culture medium, as previously described [63]. The enzymatic activity of the culture medium was measured by spectrophotometry as absorbance variance at 340 nm, and it was expressed as μmol of NADH oxidated/min/mg of cellular proteins in order to normalize the extracellular activity to the number of cells. Data were plotted relative to the untreated controls.

### 4.9. Wound Healing Assay

The assay was carried out as previously described [64], with some modifications. PANC-1 and CAPAN-2 cells were incubated with the 10 ng/mL or 2 ng/mL TGFβ for 48 h and 8 days, respectively. Then, 24 h before the end of treatments, confluent cells were starved overnight and a wound line was generated with a sterile pipette tip, followed by further treatment for 24 h. Images were obtained at 0 and 24 h using a light microscope at 20× magnification with a digital camera under bright field illumination. The area of the wound was measured in the central part of each well using the ImageJ software. The measurements were then converted into a percentage of wound closure as follows: 100 − [(area at t_24_/area at t_0_) × 100].

### 4.10. Band Quantification and Statistical Analysis

Scanning digital densitometry by ImageJ software analysis (ImageJ version 1.29, Sun Microsystems Inc., Palo Alto, CA, USA) was used for the quantification of bands from protein electrophoresis. All data were submitted to ANOVA statistical analysis with Tukey’s post hoc correction. A *p*-value < 0.05 was considered significant and indicated. Data were given as the means ± SEM of three independent experiments.

## 5. Conclusions

Our study revealed two important novel aspects of TGFβ signaling. For the first time, the interplay with vitamin D activity was unveiled in cancer cells, and it might explain the link between vitamin D deficiency and cancer risk. Moreover, we shed some light on how, in addition to the nuclear control of EMT, the different modulation of mitochondrial metabolism can tip the balance in favor of either the antitumoral or the prometastatic effects of TGFβ. A better understanding of the conditions that sustain the protumoral activity of TGFβ is of paramount importance, together with efforts to find strategies blocking the cytokine. This approach would decrease metastatic spread and reduce fibrosis, which hampers the efficacy of chemotherapy. The attempts to use anti-TGF-β-based therapies for metastatic pancreatic ductal adenocarcinoma demonstrate the importance of understanding the role of TGFβ in pancreatic cancer progression [65].

## Data Availability

All data generated or analyzed during this study are included in this published article.

## Supplementary Materials

The following are available online at https://www.mdpi.com/article/10.3390/cancers13122932/s1: Figure S1. Western blotting image source for PANC-1 cells shown in Figure 1C; Figure S2. Western blotting image source for CAPAN-2 cells shown in Figure 1C.

## References

[1] Nolte M., Margadant C. (2020). Controlling Immunity and Inflammation through Integrin-Dependent Regulation of TGF-β. Trends Cell Biol..

[2] Mantovani A., Allavena P., Sica A., Balkwill F. (2008). Cancer-Related Inflammation. Nature.

[3] Seoane J., Gomis R.R. (2017). TGF-β Family Signaling in Tumor Suppression and Cancer Progression. Cold Spring Harb. Perspect. Biol..

[4] Akhurst R.J., Derynck R. (2001). TGF-β Signaling in Cancer—A Double-Edged Sword. Trends Cell Biol..

[5] Padua D. (2009). Roles of TGFβ in Metastasis. Cell Res..

[6] Kim S.-J., Im Y.-H., Markowitz S.D., Bang Y.-J. (2000). Molecular Mechanisms of Inactivation of TGF-β Receptors during Carcinogenesis. Cytokine Growth Factor Rev..

[7] Siegel P.M., Massagué J. (2003). Cytostatic and Apoptotic Actions of TGF-β in Homeostasis and Cancer. Nat. Rev. Cancer.

[8] Siegel P.M., Shu W., Cardiff R.D., Muller W.J., Massagué J. (2003). Transforming Growth Factor β Signaling Impairs Neu-Induced Mammary Tumorigenesis While Promoting Pulmonary Metastasis. Proc. Natl. Acad. Sci. USA.

[9] Liu R.-M., Desai L.P. (2015). Reciprocal Regulation of TGF-β and Reactive Oxygen Species: A Perverse Cycle for Fibrosis. Redox Biol..

[10] Ricca C., Aillon A., Viano M., Bergandi L., Aldieri E., Silvagno F. (2019). Vitamin D Inhibits the Epithelial-Mesenchymal Transition by a Negative Feedback Regulation of TGF-β Activity. J. Steroid Biochem. Mol. Biol..

[11] Yang L., Roh Y.S., Song J., Zhang B., Liu C., Loomba R., Seki E. (2014). Transforming growth factor beta signaling in hepatocytes participates in steatohepatitis through regulation of cell death and lipid metabolism in mice. Hepatology.

[12] Sun Q., Fang L., Tang X., Lu S., Tamm M., Stolz D., Roth M. (2019). TGF-β Upregulated Mitochondria Mass through the SMAD2/3 → C/EBPβ → PRMT1 Signal Pathway in Primary Human Lung Fibroblasts. J. Immunol..

[13] Yadav H., Quijano C., Kamaraju A.K., Gavrilova O., Malek R., Chen W., Zerfas P., Zhigang D., Wright E.C., Stuelten C. (2011). Protection from Obesity and Diabetes by Blockade of TGF-β/Smad3 Signaling. Cell Metab..

[14] Dimeloe S., Gubser P., Loeliger J., Frick C., Develioglu L., Fischer M., Marquardsen F., Bantug G.R., Thommen D., Lecoultre Y. (2019). Tumor-Derived TGF-β Inhibits Mitochondrial Respiration to Suppress IFN-γ Production by Human CD4^+^ T Cells. Sci. Signal..

[15] Yoon Y.-S., Lee J.-H., Hwang S.-C., Choi K.S., Yoon G. (2005). TGF Β1 Induces Prolonged Mitochondrial ROS Generation through Decreased Complex IV Activity with Senescent Arrest in Mv1Lu Cells. Oncogene.

[16] Negmadjanov U., Godic Z., Rizvi F., Emelyanova L., Ross G., Richards J., Holmuhamedov E.L., Jahangir A. (2015). TGF-Β1-Mediated Differentiation of Fibroblasts Is Associated with Increased Mitochondrial Content and Cellular Respiration. PLoS ONE.

[17] Kawanishi S., Ohnishi S., Ma N., Hiraku Y., Murata M. (2017). Crosstalk between DNA Damage and Inflammation in the Multiple Steps of Carcinogenesis. Int. J. Mol. Sci..

[18] Snezhkina A.V., Kudryavtseva A.V., Kardymon O.L., Savvateeva M.V., Melnikova N.V., Krasnov G.S., Dmitriev A.A. (2019). ROS Generation and Antioxidant Defense Systems in Normal and Malignant Cells. Oxidative Med. Cell. Longev..

[19] Echizen K., Oshima H., Nakayama M., Oshima M. (2018). The Inflammatory Microenvironment That Promotes Gastrointestinal Cancer Development and Invasion. Adv. Biol. Regul..

[20] Krstić J., Trivanović D., Mojsilović S., Santibanez J.F. (2015). Transforming Growth Factor-Beta and Oxidative Stress Interplay: Implications in Tumorigenesis and Cancer Progression. Oxidative Med. Cell. Longev..

[21] Abe Y., Sakairi T., Beeson C., Kopp J.B. (2013). TGF-Β1 Stimulates Mitochondrial Oxidative Phosphorylation and Generation of Reactive Oxygen Species in Cultured Mouse Podocytes, Mediated in Part by the MTOR Pathway. Am. J. Physiol. Renal Physiol..

[22] Yan F., Wang Y., Wu X., Peshavariya H.M., Dusting G.J., Zhang M., Jiang F. (2014). Nox4 and Redox Signaling Mediate TGF-β-Induced Endothelial Cell Apoptosis and Phenotypic Switch. Cell Death Dis..

[23] Morry J., Ngamcherdtrakul W., Yantasee W. (2017). Oxidative Stress in Cancer and Fibrosis: Opportunity for Therapeutic Intervention with Antioxidant Compounds, Enzymes, and Nanoparticles. Redox Biol..

[24] Sawada T., Kimura K., Nishihara T., Onoda N., Teraoka H., Yamashita Y., Yamada N., Yashiro M., Ohira M., Hirakawa K. (2006). TGF-Beta1 down-Regulates ICAM-1 Expression and Enhances Liver Metastasis of Pancreatic Cancer. Adv. Med. Sci..

[25] Ye H., Zhou Q., Zheng S., Li G., Lin Q., Wei L., Fu Z., Zhang B., Liu Y., Li Z. (2018). Tumor-Associated Macrophages Promote Progression and the Warburg Effect via CCL18/NF-KB/VCAM-1 Pathway in Pancreatic Ductal Adenocarcinoma. Cell Death Dis..

[26] Deer E.L., González-Hernández J., Coursen J.D., Shea J.E., Ngatia J., Scaife C.L., Firpo M.A., Mulvihill S.J. (2010). Phenotype and Genotype of Pancreatic Cancer Cell Lines. Pancreas.

[27] Wang K., Dong M., Sheng W., Liu Q., Yu D., Dong Q., Li Q., Wang J. (2015). Expression of Vitamin D Receptor as a Potential Prognostic Factor and Therapeutic Target in Pancreatic Cancer. Histopathology.

[28] Itoigawa Y., Harada N., Harada S., Katsura Y., Makino F., Ito J., Nurwidya F., Kato M., Takahashi F., Atsuta R. (2015). TWEAK Enhances TGF-β-Induced Epithelial-Mesenchymal Transition in Human Bronchial Epithelial Cells. Respir. Res..

[29] Hosper N.A., van den Berg P.P., de Rond S., Popa E.R., Wilmer M.J., Masereeuw R., Bank R.A. (2013). Epithelial-to-Mesenchymal Transition in Fibrosis: Collagen Type I Expression Is Highly Upregulated after EMT, but Does Not Contribute to Collagen Deposition. Exp. Cell Res..

[30] Fischer K.D., Agrawal D.K. (2014). Vitamin D Regulating TGF-β Induced Epithelial-Mesenchymal Transition. Respir. Res..

[31] Silvagno F., Consiglio M., Foglizzo V., Destefanis M., Pescarmona G. (2013). Mitochondrial Translocation of Vitamin D Receptor Is Mediated by the Permeability Transition Pore in Human Keratinocyte Cell Line. PLoS ONE.

[32] Consiglio M., Destefanis M., Morena D., Foglizzo V., Forneris M., Pescarmona G., Silvagno F. (2014). The Vitamin D Receptor Inhibits the Respiratory Chain, Contributing to the Metabolic Switch That Is Essential for Cancer Cell Proliferation. PLoS ONE.

[33] Silvagno F., de Vivo E., Attanasio A., Gallo V., Mazzucco G., Pescarmona G. (2010). Mitochondrial Localization of Vitamin D Receptor in Human Platelets and Differentiated Megakaryocytes. PLoS ONE.

[34] Larriba M.J., García de Herreros A., Muñoz A. (2016). Vitamin D and the Epithelial to Mesenchymal Transition. Stem Cells Int..

[35] Stone M.L., Beatty G.L. (2019). Cellular Determinants and Therapeutic Implications of Inflammation in Pancreatic Cancer. Pharmacol. Ther..

[36] Oglio R., Thomasz L., Salvarredi L., Juvenal G., Pisarev M. (2018). Comparative Effects of Transforming Growth Factor Beta Isoforms on Redox Metabolism in Thyroid Cells. Mol. Cell. Endocrinol..

[37] Hüttemann M., Lee I., Pecinova A., Pecina P., Przyklenk K., Doan J.W. (2008). Regulation of Oxidative Phosphorylation, the Mitochondrial Membrane Potential, and Their Role in Human Disease. J. Bioenerg. Biomembr..

[38] Chen J., Tang Z., Slominski A.T., Li W., Żmijewski M.A., Liu Y., Chen J. (2020). Vitamin D and Its Analogs as Anticancer and Anti-Inflammatory Agents. Eur. J. Med. Chem..

[39] Liu W., Zhang L., Xu H.-J., Li Y., Hu C.-M., Yang J.-Y., Sun M.-Y. (2018). The Anti-Inflammatory Effects of Vitamin D in Tumorigenesis. Int. J. Mol. Sci..

[40] Pitarresi J.R., Rustgi A.K. (2019). Mechanisms Underlying Metastatic Pancreatic Cancer. Adv. Exp. Med. Biol..

[41] Bulle A., Lim K.-H. (2020). Beyond Just a Tight Fortress: Contribution of Stroma to Epithelial-Mesenchymal Transition in Pancreatic Cancer. Signal Transduct. Target. Ther..

[42] Löhr M., Schmidt C., Ringel J., Kluth M., Müller P., Nizze H., Jesnowski R. (2001). Transforming Growth Factor-β1 Induces Desmoplasia in an Experimental Model of Human Pancreatic Carcinoma. Cancer Res..

[43] Stylianou A., Gkretsi V., Stylianopoulos T. (2018). Transforming Growth Factor-β Modulates Pancreatic Cancer Associated Fibroblasts Cell Shape, Stiffness and Invasion. Biochim. Biophys. Acta Gen. Subj..

[44] Altieri B., Grant W.B., Della Casa S., Orio F., Pontecorvi A., Colao A., Sarno G., Muscogiuri G. (2017). Vitamin D and Pancreas: The Role of Sunshine Vitamin in the Pathogenesis of Diabetes Mellitus and Pancreatic Cancer. Crit. Rev. Food Sci. Nutr..

[45] Barreto S.G., Neale R.E. (2015). Vitamin D and Pancreatic Cancer. Cancer Lett..

[46] Liu M., Quek L.-E., Sultani G., Turner N. (2016). Epithelial-Mesenchymal Transition Induction Is Associated with Augmented Glucose Uptake and Lactate Production in Pancreatic Ductal Adenocarcinoma. Cancer Metab..

[47] Menezes S.V., Fouani L., Huang M.L.H., Geleta B., Maleki S., Richardson A., Richardson D.R., Kovacevic Z. (2019). The Metastasis Suppressor, NDRG1, Attenuates Oncogenic TGF-β and NF-ΚB Signaling to Enhance Membrane E-Cadherin Expression in Pancreatic Cancer Cells. Carcinogenesis.

[48] Liu Q., Sheng W., Dong M., Dong X., Dong Q., Li F. (2015). Gli1 Promotes Transforming Growth Factor-Beta1–and Epidermal Growth Factor-Induced Epithelial to Mesenchymal Transition in Pancreatic Cancer Cells. Surgery.

[49] Meyer-Schaller N., Heck C., Tiede S., Yilmaz M., Christofori G. (2018). Foxf2 Plays a Dual Role during Transforming Growth Factor Beta-Induced Epithelial to Mesenchymal Transition by Promoting Apoptosis yet Enabling Cell Junction Dissolution and Migration. Breast Cancer Res..

[50] Saxena M., Kalathur R.K.R., Neutzner M., Christofori G. (2018). PyMT-1099, a Versatile Murine Cell Model for EMT in Breast Cancer. Sci. Rep..

[51] Pavan S., Meyer-Schaller N., Diepenbruck M., Kalathur R.K.R., Saxena M., Christofori G. (2018). A Kinome-Wide High-Content SiRNA Screen Identifies MEK5-ERK5 Signaling as Critical for Breast Cancer Cell EMT and Metastasis. Oncogene.

[52] Katsuno Y., Meyer D.S., Zhang Z., Shokat K.M., Akhurst R.J., Miyazono K., Derynck R. (2019). Chronic TGF-β Exposure Drives Stabilized EMT, Tumor Stemness, and Cancer Drug Resistance with Vulnerability to Bitopic MTOR Inhibition. Sci. Signal..

[53] Schlegel N.C., von Planta A., Widmer D.S., Dummer R., Christofori G. (2015). PI3K Signalling Is Required for a TGFβ-Induced Epithelial-Mesenchymal-like Transition (EMT-like) in Human Melanoma Cells. Exp. Dermatol..

[54] Zhao J., Zhang J., Yu M., Xie Y., Huang Y., Wolff D.W., Abel P.W., Tu Y. (2013). Mitochondrial Dynamics Regulates Migration and Invasion of Breast Cancer Cells. Oncogene.

[55] Zhou H., Zhang B., Zheng J., Yu M., Zhou T., Zhao K., Jia Y., Gao X., Chen C., Wei T. (2014). The Inhibition of Migration and Invasion of Cancer Cells by Graphene via the Impairment of Mitochondrial Respiration. Biomaterials.

[56] Cunniff B., McKenzie A.J., Heintz N.H., Howe A.K. (2016). AMPK Activity Regulates Trafficking of Mitochondria to the Leading Edge during Cell Migration and Matrix Invasion. Mol. Biol. Cell.

[57] Zhang J., Zhang W., Zhang T., Zhou Q., Liu J., Liu Y., Kong D., Yu W., Liu R., Hai C. (2018). TGF-Β1 Induces Epithelial-to-Mesenchymal Transition via Inhibiting Mitochondrial Functions in A549 Cells. Free Radic. Res..

[58] Schwörer S., Berisa M., Violante S., Qin W., Zhu J., Hendrickson R.C., Cross J.R., Thompson C.B. (2020). Proline Biosynthesis Is a Vent for TGFβ-Induced Mitochondrial Redox Stress. EMBO J..

[59] Cruz-Bermúdez A., Laza-Briviesca R., Vicente-Blanco R.J., García-Grande A., Coronado M.J., Laine-Menéndez S., Alfaro C., Sanchez J.C., Franco F., Calvo V. (2019). Cancer-Associated Fibroblasts Modify Lung Cancer Metabolism Involving ROS and TGF-β Signaling. Free Radic. Biol. Med..

[60] Son J., Lyssiotis C.A., Ying H., Wang X., Hua S., Ligorio M., Perera R.M., Ferrone C.R., Mullarky E., Shyh-Chang N. (2013). Glutamine Supports Pancreatic Cancer Growth through a KRAS-Regulated Metabolic Pathway. Nature.

[61] Kalyanaraman B., Darley-Usmar V., Davies K.J.A., Dennery P.A., Forman H.J., Grisham M.B., Mann G.E., Moore K., Roberts L.J., Ischiropoulos H. (2012). Measuring Reactive Oxygen and Nitrogen Species with Fluorescent Probes: Challenges and Limitations. Free Radic. Biol. Med..

[62] Mosmann T. (1983). Rapid Colorimetric Assay for Cellular Growth and Survival: Application to Proliferation and Cytotoxicity Assays. J. Immunol. Methods.

[63] Ricca C., Aillon A., Bergandi L., Alotto D., Castagnoli C., Silvagno F. (2018). Vitamin D Receptor Is Necessary for Mitochondrial Function and Cell Health. Int. J. Mol. Sci..

[64] Loughlin D.T., Artlett C.M. (2011). Modification of Collagen by 3-Deoxyglucosone Alters Wound Healing through Differential Regulation of P38 MAP Kinase. PLoS ONE.

[65] Alvarez M.A., Freitas J.P., Mazher Hussain S., Glazer E.S. (2019). TGF-β Inhibitors in Metastatic Pancreatic Ductal Adenocarcinoma. J. Gastrointest. Cancer.

