# Supplementary material for: TGF Beta Induces Vitamin D Receptor and Modulates Mitochondrial Activity of Human Pancreatic Cancer Cells
Source: Cancers (Basel). 2021 Jun 11;13(12):2932. doi: 10.3390/cancers13122932 (PMC8230851; doi:10.3390/cancers13122932)
Supplement: Supplementary file 1 [file cancers-13-02932-s001.zip › cancers-1256018-supplementary.pdf]

cancers

Supplementary Materials

TGF Beta Induces Vitamin D Receptor and Modulates Mitochondrial Activity of Human Pancreatic Cancer Cells

Camilla Fiz, Giulia Apprato, Chiara Ricca, Alessia Aillon, Loredana Bergandi and Francesca Silvagno

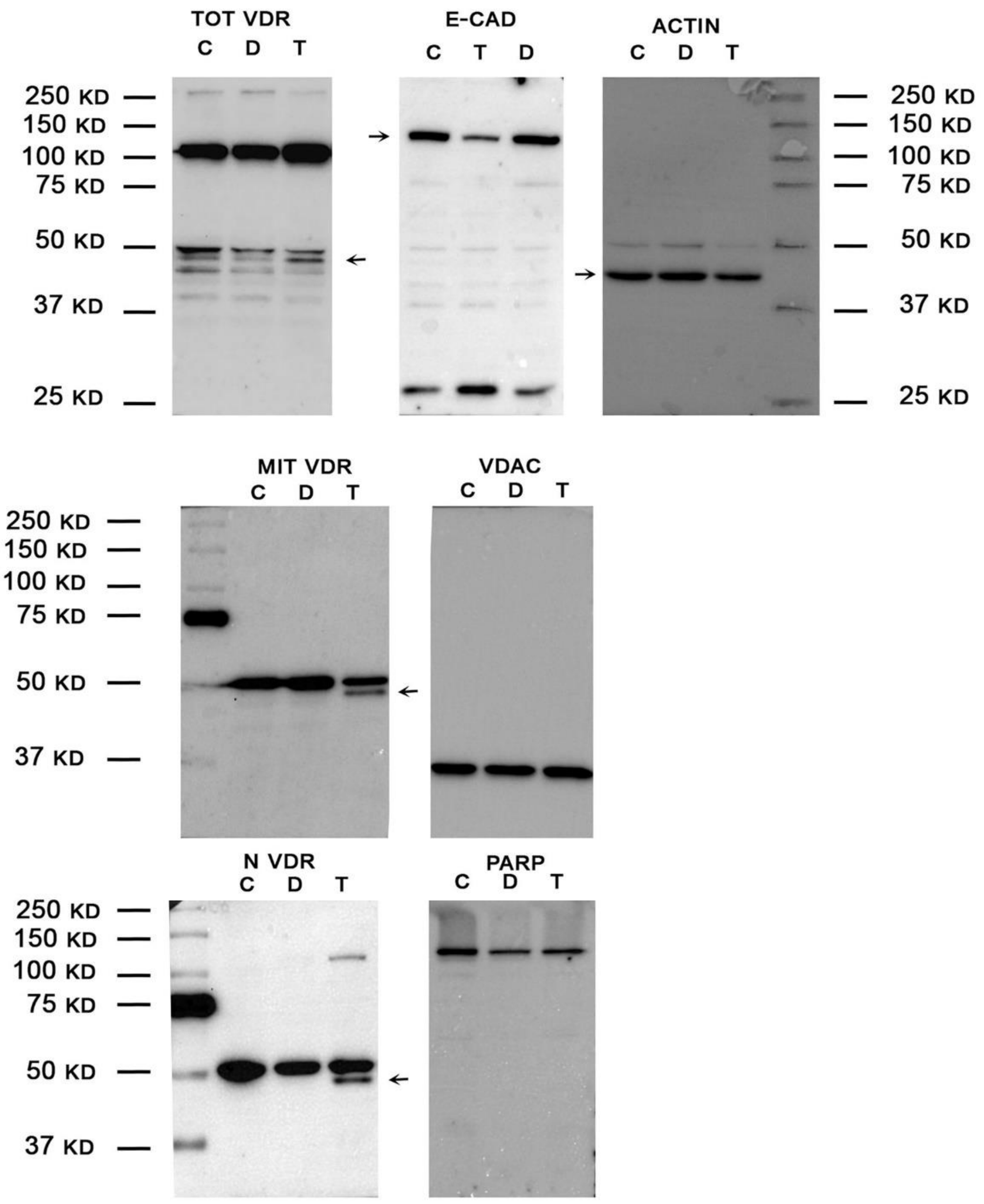

Figure S1. Western blotting image source for PANC-1 cells shown in Figure 1C.

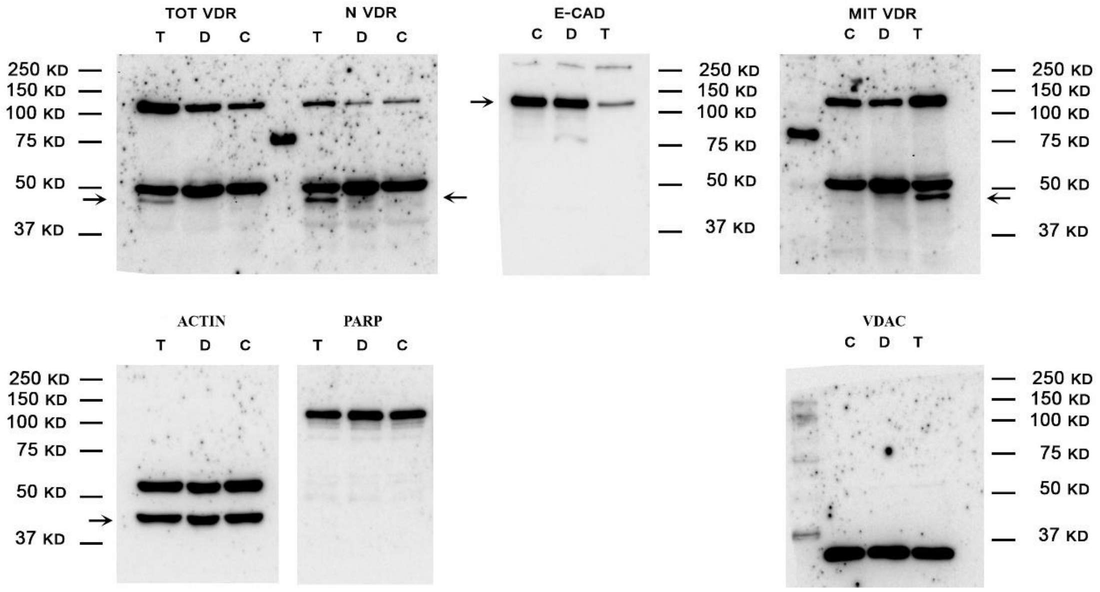

Figure S2. Western blotting image source for CAPAN-2 cells shown in Figure 1C.
